# Discussions before the Twentieth Annual Meeting of the Indiana State Dental Association, June 29th, 1870

**Published:** 1870-09

**Authors:** 


### Proceedings of Societies.

---

### DISCUSSIONS BEFORE THE TWELFTH ANNUAL MEETING OF THE INDIANA STATE DENTAL ASSOCIATION, JUNE 29th, 1870.

### FIRST DAY, AFTERNOON SESSION.

Dr. W. F. Morrill read a carefully prepared essay on the subject of Hypodermic Medication.

The discussion of this subject was deferred for the introduction of a subject upon mechanical dentistry.

Dr. Keightley opened the discussion on "The Best Substitute for Vulcanized Rubber as a Base for Artificial Teeth."

The Doctor described a process of his own, not yet patented, for the preparation of aluminum, which metal he admires very much as a base for artificial teeth.

Dr. Femster, of Greencastle, also described his process, not yet patented. He found out by experiment that the proper heat is about eight hundred degrees; the reservoir should be red hot, between nine hundred and one thousand degrees. He exhibited a base which he borrowed from a lady for the purpose, that had been worn about four months. Since commencing the use of aluminum he made but two failures in twenty-five cases. In about fifty minutes after the fire is kindled, the metal is ready to inject. It takes from one to two hours to cool. He preferred two hours. Since completing his experiments he has been more universally successful in making fits with his metal than with rubber. He finds it stands the influences which were expected to operate against it very well, and regards it as the best substitute for rubber, in all probability, that can be gotten, and he hoped to see the time when it will be universally used.

This new metal happily compensates for all the difficulties heretofore met by the profession. He promised to supply aluminum at moderate figures, and guaranteed an article that shall be pure. He thought orders of twenty pounds could be filled at ten dollars a pound, and was under the impression that in five years the price would fall to three dollars a pound. He illustrated his process of casting by drawings upon the black-board.

Prof. Watt, of Cincinnati, was much gratified at the results in working aluminum; it is so much better than the rubber nuisance. He was of the opinion, however, that it would not prove to be entirely satisfactory. He commenced experimenting with it in the spring of 1856, and spent a considerable portion of the summer at it. In mouths containing chlorine, the aluminum undergoes pretty rapid corrosion; and if you crack it in swageing, you are annoyed much more than with a gold plate. Last March a year ago he commenced a series of experiments, with a view to get something more easily manipulated than aluminum—so simple as to induce even the tyros to abandon the use of rubber, and he thought he had succeeded in obtaining a metal that is about as easily cast into dental plates as lead is into bullets. It is known as Watt and Williams' improved alloy, and will resist chloridations much better than aluminum or silver, and as well as 18 carat gold, and sulphidation as well as 19 carat gold. Dr. Hendrick has had a plate of this metal in his mouth for two weeks, which remains as bright as the day it was made, while he has tried even coin gold, and it corrodes. This alloy successfully resists sulphur, oxigen and chlorine, the three pirates that run against the dentists.

He exhibited specimens, and claimed that this metal would be found much more satisfactory than aluminum for under sets Those who find it too heavy for upper sets will find another substitute for rubber in a combination of platinum and pure silver, very nearly the color of platinum. If we have succeeded in getting something so simple, that will answer the purposes of rubber, mankind will be benefited by the change, for he is convinced that the rubber nuisance ought to be promptly abated, as humanity has been poisoned long enough with that infernal nuisance.

Dr. Femster has noticed this new work with deep interest, and had labored with zeal, but had never seen any substance in nature, in all the sixty-nine elements, so admirably adapted as a base for artificial teeth as aluminum. Its lightness, strength, etc., renders it peculiarly adapted to that purpose. A process to cast directly upon the teeth has been a great desideratum with the profession. If we get any thing that is superior to aluminum, we have got to have it as light as aluminum, and as easily worked. There is nothing so perfectly inert as aluminum.

Dr. Stanley spoke a word in favor of this alloy, alleging that it retains its color, —a light silver—better, if any odds, than aluminum.

Prof. Judd, of St. Louis, is as anxious as any one to see rubber go out of use, for several reasons, the first and greatest being because he is perfectly satisfied it is poisoning thousands of people annually. He made this statement advisedly, after examinations that have satisfied him of its perfect correctness; and he thought most of the best men of the profession have come to the same conclusion. It is, as has been declared, of great importance to find a substitute for rubber, that we have one equally cheap and as easily manipulated. We all acknowledge the force of the argument made on this point, viz: any agent that is brought generally into practice must be cheap and easily manipulated. He believed, from the experiments he had, even with aluminum, that it will stand the action of the fluids of the mouth perfectly well in a vast majority of cases. Strong mineral acids, except chloric, do not affect it, but the amount of that acid in the mouth is exceedingly small where there is any of it, and experience demonstrates that in a great majority of cases it retains its color for many months, and he had seen it remain for years. He fully subscribed to the truth of the doctrine that in certain cases it does change in the mouth, where there is a definite amount of hydrochloric acid; but as a general thing, in a vast majority of tests, it stood better than any thing he had ever seen in the shape of metal, except gold and platinum. This last he hoped would answer the purpose, and should be exceedingly glad to have any thing brought forward, and will encourage the use of such as will answer the purpose. He thought that aluminum was the easiest to swage of any metal except lead, and he would hardly even except lead. In one hundred plates he never broke one. This method of casting plates seems to fulfill all requirements. There are several other patents for working aluminum. One gentleman in Carthage, Ill., has a patent for casting. Dr. Lawrence, of Lowell, Mass., has one. A great many are alarmed at the idea of machinery, and he had no doubt but that would drive off many from attempting the use of aluminum. In regard to platinumizing silver, he had not seen any of it, but in his city they have been platinumizing other metals, and as far as protecting the metal from the juices of the mouth is concerned, it seems to work admirably. It is an exceedingly simple process, and it has been stated by some that it could probably be done for twenty-five cents apiece. One man in St. Louis makes a business of it, but he asks one dollar apiece. He had been comparing these metals with rubber. He did not suppose that any of these base metals will fulfill all the indications gold and platinum do, but that they would prove vastly superior to rubber in all shapes and forms, he had no doubt whatever.

Dr. Morrill stated his observations of the aluminum plates to be that where they are worn any length of time there is a deposit on them of lime or other secretions from the mouth, even where particular care has been exercised. As a substitute for rubber he recommended rose pearl. It can be made more thin; it is lighter than aluminum, though it is light enough, perhaps too light, and as to color it is a close imitation of nature. There is hardly any thing, in his judgment, that comes up to rose pearl in this particular. And as far as cheapness is concerned, it is very near the same as rubber. Many first class dentists are using it, and it is bound to come into general use sooner or later.

In those cases where the gums are protruding—what he called bell-shaped gums, you can carry this material over the border as thin as paper, and it has great advantage in the facility with which the edges can be moved—if you wish to change slightly the edges of the plate, you can do so, which you can not do with aluminum. It is well known to every Dentist that there are hardly any plates of metal of any kind but have to be changed at some time or another. He thought that if the Dental profession would turn their attention to rose pearl, they would find in it many advantages, and believed it is a material that is bound sooner or later to be used, for it is a material that has advantages over any other substance yet used. In reference to an inquiry as to what length of time it takes to prepare this substance, Dr. M. replied, thirty-six hours; that is, the time necessary for the evaporation of the solvent.

On motion the discussion was closed on this subject.

Dr. Knapp being called upon, read his essay, entitled "What are the Indications for Destroying the Pulp?"

The Association proceeded with the discussion of the subject of Dr. Knapp's paper.

Prof. Taft, of Cincinnati, contended earnestly for the saving of the pulps, or nerves, of the teeth. This subject ought to receive more attention from the profession. It is difficult to properly apprehend it without a thorough knowledge of some general principles that will reach out to other things as well as this. An answer to the question under consideration involves a good deal. The indications are not always the same. There are many circumstances that will vary the indications. In the first place the conditions of the patient in regard to temperament and health will modify the course of treatment that should be pursued towards an exposed pulp. No two persons physically have the same susceptibilities. Some, for various causes, are susceptible to influences others would not feel at all. The living tissue of the body will be very much injured in one case that in another would scarcely be felt. Inflammation will spring up in one person from a slight scratch or wound that another would scarcely feel and not notice an hour afterward. This difference of susceptibility we must regard in every case. The pathological condition of the person must be taken into account. Then the exposure of the pulp itself; the influences brought to bear upon it since its exposure; whether recently or a long time exposed; also, the location of its exposure, etc. Then the anatomical character of the tooth itself should be considered. And in order to treat the teeth successfully, it is necessary to have an accurate knowledge of their formation. In addition to all these things, and many others we might name, the operator should have confidence in his knowledge and ability in every direction. His manipulative skill and understanding constitute so many conditions as to what would be the proper course of treatment for him to pursue. It is not proper for all operators to pursue the same course. One course of treatment may be indicated clearly to one person, while quite a different one should be pursued by another. There are skillful operators who do not succeed in saving the pulps of teeth ordinarily as well as men with far less skill and knowledge, because the latter having stronger faith, will and determination to accomplish the object, make every thing over which they have control bend to their purpose. If we will accomplish anything we must have faith in our ability, and then proceed and use the highest skill we possess. If one attempts anything without faith and confidence in his ability —whatever that ability may be—the probability is that failure will be the result. There is a power and influence in faith and determination beyond and above mere physical effort. The indications for destroying the pulp are not now to me what they were years ago. Then I destroyed a great many pulps, but am continually undergoing a change. I have been and now I expect to keep changing as long as I live. If I ever get to a point where I make no change, then I think it will be time I should go. Formerly I destroyed a great many pulps of teeth that I now save. Circumstances now make this work far easier than formerly. We have therapeutic agents that act in a way that we could not formerly understand. Our knowledge has vastly increased within a few years. The exposed pulp should be placed back again as nearly as possible to its original condition and position. The pulp is shut up in its bony walled house. It occupies all the space, and the space is just big enough for it. We did not then have agents to accomplish the object properly. It has remained to these latter times to furnish the material which fulfills the requirements. After placing the pulp back as nearly as possible to its original position, there is no more difficulty in saving the pulp alive, than in arresting dental decay by filling. The indications for the destruction of pulps of teeth are now rare. Some times so low do the vital forces become that the teeth die spontaneously.

There should usually be but little interference with the teeth during times of great vital depression. In such cases first impart strength and tone to the system, and secure all the advantages that may be derivable from such a condition. After that is accomplished, go to work carefully upon the teeth, always saving the pulps alive if possible. A great fault is that we too much ignore all these things, and don't try to examine and work through the whole subject.

Prof. Watt supposed this question can never be settled absolutely and definitely. We will have to go upon general principles, as the surgeon can only approximate as to the indications for amputation in compound fractures. He did not think there was much difference in the pathology of an exposed pulp and a compound fracture. Salvation is emphatically the gospel of the West. What light there is in the East has been transplanted from the West. The Eastern slope was full of destructionists. There had been no uniformity of sentiment on that subject, but now almost every one recognizes the doctrine, which received a great impulse from the late war, that the general surgeon endeavors to save the limb where there is a compound and comminuted fracture. Yet a few years ago any surgeon would have been prosecuted for malpractice if he even made the attempt to do what is now an every day accomplishment. He knew of cases of pulp salvation so remarkable that it would be almost unsafe to tell what had been done in that direction. But the point he desired to make was this: Those men connected with the profession who expect recipes to tell them in such a condition the pulp can be saved, and in such a condition it must be destroyed, instead of going by their own brain power, are not likely to succeed. He concluded by advising the practitioner to give the patient the benefit of the doubt; if you have a little doubt, try to save it; when you find you can not save it, destroy it.

Prof. Judd, of St. Louis, did not know that he could point out any case where he could say it would be proper to destroy the pulp. The ground has been covered by the discussion already. It is difficult to discuss a question where all are on one side. All want to save every pulp we can. There are some indications that might lead us to destroy a pulp. In cases of long continued inflammatory action, there are changes taking place in the tissues that are inflamed. Some cause atrophies, hypertrophies, and we find in some instances, of hyperfacia, and an increase, not in the elements themselves, but in the number of elements; a large cavity filled, and all of this abnormal structure, and still alive. It is a living tissue, filled with vessels, and crowded in a great many instances with blood. In such a case as that we would say we have an unmistakable case in which it would be proper under all circumstances to destroy the pulp. Prof. Judd holds that it is impossible to save a tooth after the pulp has been extirpated. This has been disputed by modern authors, and there are many cases in which they have been saved after extirpating the pulp. And there is a great difference in filling the fangs of teeth and in filling teeth themselves. He would not extract a tooth till he had tried by extracting the pulp and filling the tooth to save it.

In the preservation of pulps he had been very successful. It has been objected to by many that these pulps die and give rise to disease. I remember that a Dentist stated that in the last three or four years he had covered pulps in fifty cases, and he found over twenty had given rise to disease. That may be true. I am not disposed to question the gentleman's word. But I am sure he has not been as fortunate as most of those who have made use of these means.

Prof. Taft was of opinion that such a statement goes to prove the man did not know what he was about; did not give the proper kind of treatment. A great many pulps are covered up hurriedly that ought to have received more careful attention. This is one of the most delicate operations we have to perform, and requires the most delicate and thorough diagnosis. The treatment should be of the most delicate character. The operation should be just right, and when all these indications are fulfilled, in nineteen cases out of twenty success will follow. He could not remember a case in his practice where disease followed.

Dr. J. F. Johnson did not believe any one mode of practice will always be successful, but thought one of the indications for the destruction of a pulp was when the patient had suffered severely and for a long time from it, the Dentist being unable to afford relief. He did not pretend to torture the patient with further delays to keep it, but would immediately apply the arsenical paste. Where a pulp with slight covering is presented to him, he removes that and covers it perfectly. Just the other day he had a patient with nice teeth who had been suffering with an exposed pulp. Although only a small cavity, she had suffered several days with a soreness on pressure, and the gums were inflamed. He cleansed it very carefully and filled the tooth with oxy. chloride of zinc, and it remained quiet for a time, when she came back with a bad toothache, and he had to take that out and destroy the pulp. Had the condition been understood, he would not have tortured the woman, but applied the arsenical paste at once.

Prof. Taft spoke of another condition. When the pulp has been exposed for a considerable length of time, and been inflamed, and there is inflammation of the periosteum—where there is that complication he did not think the pulp could ordinarily be saved. But that does not often occur—periostitus and an inflamed pulp at the same time. Such cases are the most difficult to manage. Before resorting to arsenious paste in cases of that kind, would endeavor to remove the pulp by an operation. He fears the influence of arsenic paste on it, for he had seen two cases in which the entire loss of the tooth took place. Where there is calcification of the pulps, there will be found little granules of calcific material occupying the upper part of the chamber; the upper part of the pulps will be converted into a granular mass, press upon that beyond, and pain will be experienced. Formerly he endeavored to remove this calcified part, which gave the patient a great deal of annoyance; but for a considerable length of time he had not removed these at all, but simply removed all loose matter, then put in pledgets, moistened with creosote, cover this with *os-artificiel*, then fill with gold. He did not know whether that was the best way or not, but as yet he knew of no trouble having occurred in such cases.

Dr. Johnson remarked further of the case to which he had referred, that after removing the pulps and applying the arsenical paste, all inflammation disappeared and the patient was at perfect ease. Then she had a good deal of pain of a sympathetic nature. He took it for granted that Prof. Taft's theory is good, that the blood let in drawing the nerve was beneficial, but it is such a painful operation that female patients especially shrink from it, unless put under the influence of an anæsthetic, which he did not think advisable.

Prof. Taft, in referring again to cases where the pulp has been inflamed for a considerable length of time, remarked that that of itself is not an indication for its removal. If the patient were in good health he would make an effort to save the pulp. He would apply anti-inflammatory preparations, and he knew he had succeeded in saving such cases. Then he contended the removal of the pulp by an instrument is not so formidable as many suppose. He would not run a barbed instrument into it, but a thin, smooth instrument, with sharp, slightly curved, cutting edge, that will not wound the pulp. Run it up to the end of the root, then turn it, and you will find the pulp cut off, in many cases giving the patient no pain. Some times it gives pain. Put cotton, saturated with chloroform, in the cavity for a few moments. Ordinarily he found no difficulty in removing pulps by an operation.

Dr. Johnson inquired whether Dr. Taft knew of any indication which can be relied on in every case for the destroying of a pulp.

Prof. Taft replied, only the cases Dr. Judd spoke of and the complications he had referred to. It is almost impossible to define exactly the cases where the pulp should be removed.

Dr. Knapp thought the question must be settled by each individual for himself. When we have done all that we can do, or all that we can get the consent of the patient to let us do, and the case grows worse, we should destroy the pulp. He had tried but one or two in which he had failed. We have a difficulty in the northern part of the State from the influence of malaria, and some people's pulps become so inflamed from this influence and so painful that it is difficult to persuade the patient to forego extraction. He had said to some, "Positively, I will not take that tooth out," "Or if you are going to have that tooth pulled, go somewhere else."

Prof. Taft.—It may be said that as we do not arrive at any conclusion, therefore all this talk don't amount to much; but it does amount to something, for we can't talk about these points without learning. We can gain knowledge; we can stimulate one another to go on and do better; and in many other ways these discussions do good. He thought he was learning faster and more than ever before. There is no better way to learn than to come together and consider these things, and the brethren ought every one to take part. He never went to these meetings that he did not learn much, and in addition became stimulated to do more than ever before.

Dr. Burgess wished Dr. Taft to tell how he proceeded in case a person comes in with an aching tooth and you do not extract it nor destroy the nerve.

Prof. Taft.—Where the patient is in a good condition otherwise, and there is simple inflammation of the pulp, examine it, clean out the cavity, free it from every impurity, then some times simply apply creosote; if the pain does not cease, apply carbolic acid and glycerin, some times spirits of camphor; some times nothing but glycerin. A variety of things may be applied. He did not remember a case of simple toothache he had not relieved in a few minutes. But there may be cases where there is a condition existing in which we can not thus readily attain success. Where everything goes favorably for three or four days, the tooth may be filled. Should the pulp be exposed and give some indications of pain, use occasionally nitric acid, touch to the point exposed, and go on and fill over that. The use of acetic acid in that way has been very successful. Some times chloroform will answer every purpose.

Prof. Moore, of Lafayette, acknowledged Dr. Taft to be good authority; but after naming over his own experience, while sitting here, he could hardly think of an indication that would justify the destruction of a pulp.

Dr. Morrill, of New Albany, could not exactly agree with Dr. Moore, and could conceive of one indication where it is advisable to destroy a pulp, and that is where inflammation has taken place, and the patient gets in a condition in which the pulp is apparently sloughing away; where, when you examine it, it seems from probing around that the dead portion of the pulp is attached to the living portion at some point in the chamber or canal portion. If we should try to save more it would be better. He has lost but three or four cases. We can not always get at the exact truth from patients as to how much previous inflammation or irritation has taken place.

Dr. Rawls, of Connersville, did not exactly agree with Dr. Morrill's assertion in reference to a pulp, part of which is dead. He did not believe it necessarily follows that the remaining portion should be destroyed. We know that diseases of the body everywhere admit of treatment; the line of demarkation between the healthy and unhealthy part being on the external portion of the body. The operator should always give the patient the benefit of all doubts in these cases. As regards the indications for filling teeth he had found that in nine cases out of ten where the patient complained of pain at the time of the application and a chilly sensation, he had a successful case and could go on and fill the tooth immediately. But where the pain does not come on after five minutes, in nine cases out of ten the treatment had better be renewed. Where there is sharp pain following the application of chloride of zinc, nine out of ten will prove successful. We don't understand enough about the diagnosis of disease and therapeutics in general.

Prof. Taft.—There is too often a want of a just appreciation of the importance of preserving the life of a tooth. It should be remembered that when the pulp of a tooth is destroyed, its vitality is destroyed, and that the tooth is certainly lost much sooner than if the vitality remained. There are none of us who regard our teeth with the sacredness we ought.

Prof. Judd was glad to hear Dr. Taft speak in this way, for he had been talking in this way for some time himself, and had been taken to task for it. The reason of his earnest desire to preserve the pulp is because he believes that the teeth are vital structures.

The President declared the next question, the fourth on the list, to-wit: "Filling Teeth with Gold." Prof. A. M. Moore, of Lafayette, to open the discussion by reading a paper on the subject, which see in the August number of REGISTER.

The discussion of the subject being now in order, Prof. Judd insisted that a good method is to put in crystal gold to start on. He had used it off and on for a good many years. Then the drilling of retaining points is an important matter. Take a tooth which is exceedingly sensitive, and in such a case the drilling to make a foundation is no small item. In cases like that he had invariably made use of Morgan's plastic gold for purposes of making a foundation, and he wanted no points drilled. But he advised being exceedingly cautious about handling it. Lay it carefully, and work it slowly with an instrument until the foundation is well cushioned all over, then mallet it down. Let there be enough plastic gold, so that the instrument will not pass through it, and you will have a foundation that will never slip. If you get the method of it, you will never fail. In regard to the kind of gold for filling up, for several months past he had used not less than No. 20. He had used from some ten different manufacturers within about eight months, and found a good many good, but some totally unfit for use. He thought he had the first book of No. 60 that has been manufactured for many years, manufactured expressly for his own use, and he was sure he had the first book of No. 120, manufactured expressly for his own use. With the present light he had, he preferred No. 30 to anything he had used in this line. Get No. 20 to No. 30 for large cavities, crumple them up and place in the whole at once. He uses the smallest points for this. It is necessary to use tolerably small points, for the foil is very heavy. This gold is not as adhesive as the thicker, and it requires less amount of force to fill a tooth with heavy foil than with light foil.

Prof. Taft knew of nothing better, in general, out of which to make a foundation for filling, than crystal gold. His method is to use the lighter foils; the extent and manner depending somewhat upon the size, form and location of the cavity to be filled. He would fill a cavity three-fourths or four-fifths in this manner, being careful to have the gold well attached to the walls of the cavity, so that if the surface were to come off, moisture would not get down the walls of the cavity. Then take No. 60, cut the pieces a little larger than the orifice of the cavity, and lay on one and another, condensing each as put on. By this means you get a thorough adaptation of the foil to the walls of the cavity. He had used No. 120, but uses No. 60 more. He did not know but that Dr. Judd's method was quite as good, or there may be better methods.

Dr. Hollingsworth, remembering that Prof. Taft referred to the use of a small pointed instrument, desired to know the character of the instrument Prof. Judd uses.

Prof. Judd replied that he used a larger instrument than Dr. Taft, with round points, the same general form, but rather larger. When using heavy foil he wanted finer instruments. The instrument should be as large as can be used to consolidate the gold and have the tooth bear the force necessary to accomplish that. It evidently requires more time with a fine point than with a larger one, but more force is necessary with a large one than with a fine one. In a good tooth, in a strong, robust person who can bear it, we can drive it home rapidly with a mallet.

Dr. Hunt desired to know if any one present had experimented with rolled gold—gold that has not been hammered at all? He had used it, and could not see but that it made as good fillings as heavy foil that has been hammered. He desired to know whether there is anything peculiar in gold that has been beaten; whether it is more adhesive or not.

Prof. Judd replied that rolled gold has been the rage at St. Louis for about two months. They have hardly used anything else. He spoke of three other Dentists there beside himself who had used it extensively, and he believed all but himself have stated that it did work better than any beaten gold they ever used. He had received a letter from Dr. Cushing, of Chicago, speaking very highly of its qualities; but still he was not yet converted to the doctrine that rolled gold work is better than the other.

Dr. Taft expressed the opinion that rolled gold acts just as well as foil.

The valedictory of Dr. J. F. Johnston, of Indianapolis, retiring President, was of the character usual to such occasions. After expressing his appreciation for the thrice conferred honor of an election to the chair, he alluded to the unnecessary habit of lecturing the profession upon good breeding and morals, taking it for granted that these are a prerequisite upon entering it. He alluded also to the necessity for a State law to regulate dentistry in Indiana; congratulated the association upon the progress made during the twelve years of its existence, yet cautioned it in the matter of admissions without a thorough investigation of the character and standing of applicants.

Dr. Johnston was clearly of opinion that the establishment of a Dental journal would greatly advance the interests of the Association and the profession, and thought that it could be made not only self-supporting but profitable. In that connection he believed that the publication of a list of the members in good standing would exert a wholesome influence and heighten the value of the journal.

